# BOLD Frequency Power Indexes Working Memory Performance

**DOI:** 10.3389/fnhum.2013.00207

**Published:** 2013-05-16

**Authors:** Joshua Henk Balsters, Ian H. Robertson, Vince D. Calhoun

**Affiliations:** ^1^Trinity College Institute of Neuroscience, School of Psychology, Trinity College DublinDublin, Ireland; ^2^Neural Control of Movement Lab, Department of Health Sciences and Technology, ETH ZurichZurich, Switzerland; ^3^The Mind Research NetworkAlbuquerque, NM, USA; ^4^Department of Electrical and Computer Engineering, University of New MexicoAlbuquerque, NM, USA

**Keywords:** BOLD oscillations, ICA, fMRI, delayed match-to-sample, aging

## Abstract

Electrophysiology studies routinely investigate the relationship between neural oscillations and task performance. However, the sluggish nature of the BOLD response means that few researchers have investigated the spectral properties of the BOLD signal in a similar manner. For the first time we have applied group ICA to fMRI data collected during a standard working memory task (delayed match-to-sample) and using a multivariate analysis, we investigate the relationship between working memory performance (accuracy and reaction time) and BOLD spectral power within functional networks. Our results indicate that BOLD spectral power within specific networks (visual, temporal-parietal, posterior default-mode network, salience network, basal ganglia) correlated with task accuracy. Multivariate analyses show that the relationship between task accuracy and BOLD spectral power is stronger than the relationship between BOLD spectral power and other variables (age, gender, head movement, and neuropsychological measures). A traditional General Linear Model (GLM) analysis found no significant group differences, or regions that covaried in signal intensity with task accuracy, suggesting that BOLD spectral power holds unique information that is lost in a standard GLM approach. We suggest that the combination of ICA and BOLD spectral power is a useful novel index of cognitive performance that may be more sensitive to brain-behavior relationships than traditional approaches.

## Introduction

Studies of neural oscillations are pervasive in neuroscience, from single and multi-unit recordings through to non-invasive whole brain methods such as electroencephalography (EEG) and magnetoencephalography (MEG). Studies using these methods have repeatedly demonstrated that the synchronization of neural oscillations within specific frequency bands impact on cognitive and motor processes (Klimesch, 1999; Buzsaki and Draguhn, 2004). For example, a number of studies have highlighted the role of mid-frontal theta in cognitive control (Cavanagh et al., 2009; Cohen and Cavanagh, 2011), whilst posterior alpha power has been linked to sustained and spatial attention (Thut et al., 2006; Dockree et al., 2007; O’Connell et al., 2009). Nearly 20 years ago Jezzard et al. (1993) and Biswal et al. (1995) demonstrated regional BOLD differences in low frequency oscillatory fluctuations (0.01–0.1 Hz). Since then a large number of studies have demonstrated that this <0.1 Hz BOLD signal relates to underlying neural processes (He et al., 2010; He, 2011; Honey et al., 2012) and can be used to detect differences in resting connectivity between clinical populations (Greicius et al., 2004; Jafri et al., 2008; Zhang and Raichle, 2010), as well as task-related changes in functional networks (Grady et al., 2010; Zhang and Li, 2012). However, these aforementioned studies have used spectral information as a filtering tool, typically removing signal >0.1 Hz in order to remove potential artifacts, rather than analyzing the relationship between BOLD oscillations and task performance as one might in an EEG or MEG study. To our knowledge no previous studies have investigated whether a direct correlation exists between task performance (i.e., accuracy) and BOLD spectral power at different frequencies.

To date it is mostly resting state studies that have investigated BOLD oscillations. Studies investigating BOLD oscillations at rest have demonstrated that multiple frequency bands within the 0.004–0.15 Hz range contribute to the RSN signal (Niazy et al., 2011). Niazy et al. (2011) also showed that phase synchrony differs within this spectral range, suggesting that RSNs likely contain multiple oscillatory components. Studies by Baria et al. (2011) and Zuo et al. (2010) have additionally shown that BOLD signals originating from different cytoarchitectonic and anatomical regions resonate at distinct frequency ranges. Baria et al. (2011) is one of the few studies to also investigate BOLD oscillations during task performance (visual-motor task). They found a global decrease in lower BOLD frequency oscillations (0.01–0.05 Hz) during task compared to rest along with a global increase in higher frequency BOLD oscillations (0.05–0.1 Hz). Compared to a standard general linear model (GLM) analysis there was less than 30% spatial overlap in regions showing task-related differences in BOLD oscillations, suggesting that BOLD spectral changes are not detected by standard fMRI analyses. Salvador et al. (2008) investigated connectivity within the frequency domain [differing from Baria et al. (2011) who investigated regional changes in BOLD spectral power] and found increased low frequency connectivity (<0.08 Hz) between prefrontal, parietal, and thalamic regions during performance of an N-back task compared to rest. There was also decreased high frequency connectivity (0.08–0.25 Hz) during the N-back task within the anterior cingulate/paracingulate gyri and insula. Whilst both Salvador et al. (2008) and Baria et al. (2011) have shown that BOLD oscillations differ in task compared to rest conditions neither of these studies investigated the extent to which task performance was correlated with BOLD spectral activity.

Apart from Baria et al. (2011) and Salvador et al. (2008) no other studies to date have investigated the relationship between BOLD spectral power and task performance. However, a handful of fMRI studies have begun to investigate temporal variability within the BOLD signal and its relationship to task performance. In a series of studies by Garrett et al. (2010, 2011, 2013) they used a partial least squares approach to extract functional networks and subsequently analyzed the variability (standard deviation) within these circuits and their relationship to age and task performance. Garrett et al. (2010) showed that BOLD variability was a robust marker of chronological age, explaining more age-related variance than mean BOLD signal. BOLD variability was also an important indicator of task performance. Garrett et al. (2011) showed that young participants increased BOLD variability during task performance and decreased variability during fixation. However, elderly participants failed to modulate BOLD variability between task and fixation conditions, showing reduced variability during task and increased variability during fixation. Samanez-Larkin et al. (2010) used a similar analytical approach and demonstrated increased BOLD variability in elderly participants within the nucleus accumbens (NAcc), which was associated with increased financial risk taking. As with the work of Garrett et al. (2010, 2011, 2013), Samanez-Larkin et al. (2010) found that these results were specific to BOLD variability measures and that the average NAcc signal did not predict risk seeking behavior. It is clear from both of these studies that BOLD variability might be a more sensitive measure of functional changes with age than average BOLD signal. It is likely that these changes in BOLD variability have an oscillatory underpinning and could be better explained by investigating the BOLD spectrum.

The previously mentioned studies show that spectral properties of the BOLD signal are anatomically and functionally informative, although this approach has typically only been applied to resting state fMRI. Studies investigating BOLD variability during task performance suggest that this measure holds unique task dependent information that is lost in a standard GLM analysis. Using tools available in the GIFT toolbox, we aim to bridge the gap between studies of BOLD oscillations at rest and studies of BOLD variability during task by investigating the relationship between BOLD spectral power and task performance (delayed match-to-sample task) in young and older participants.

## Materials and Methods

### Participants

Sixteen young (22.08 ± 3.31) and nineteen elderly (70.2 ± 3.96) neurologically normal, right-handed subjects participated in this study. The two participant groups were matched for gender, handedness, hospital anxiety and depression scale (HADS) score, and Mini Mental State Exam (MMSE) score. Participants gave written informed consent prior to the study that was approved by the Trinity College Dublin School of Psychology Ethics Committee.

### Procedure

#### Trial structure

**Figure 1:**
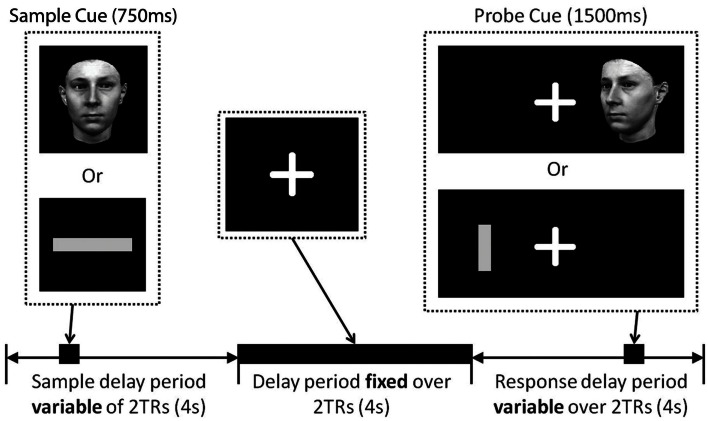
**Delay match-to-sample trial structure**. A fixation cross was presented in the center of the screen for the duration of the study. Sample cues (either a face or a line) were presented in the center of the screen during the first 2TRs of a trial (stimulus onset jittered 0–3250 ms from the onset of the first TR), after a variable time delay (4299–9630 ms), a probe cue was presented left or right of the fixation cross (stimulus onset jittered 0–2500 ms from the onset of the fifth TR). Participants responded as quickly possible at the presentation of the probe cue making either a left/right judgment or a match/non-match judgment. Face and line probe cues were presented in a different orientation to the sample cue.

Figure 1 illustrates the trial structure. Throughout the experiment participants were asked to fixate on a white cross hair presented in the center of a black screen. The same basic trial structure was applied in all conditions, with condition-specific variations (see “Conditions” below). After a variable inter-trial interval (1782–6881 ms) a sample cue was presented in the center of the screen for 750 ms. This was replaced by the crosshair for a variable period between 4299 and 9630 ms. A probe cue was then presented left or right of the cross hair for 1500 ms. At this point the participant made a judgment about the stimuli by pressing the left or right button on the keypad placed in their right hand. No feedback was given to the participant about their response. In all trials the probe stimulus was presented at a different angle/orientation to the sample stimulus so they were not perceptually identical. This forced participants to encode stimulus identity and not just perceptual features of the stimulus.

#### Conditions

Four trial types were embedded in a 2 × 2 factorial design (two factors each with two levels).

##### Factor 1: task (match, respond)

Participants performed four blocks where they had to make a judgment about whether the sample and probe matched (Match) and four blocks where they had to make a judgment about the position of the probe (Respond). These blocks were pseudo-randomly intermixed. At the beginning of each block a cue was presented for 750 ms saying “MATCH” or “RESPOND.” This informed the subject which task they had to perform for the block. In blocks of Respond trials participants responded by pressing the left button if the probe cue was on the left of the screen, or the right button if the probe cue was on the right side of the screen. During Respond trials participants did not need to encode or attend to the sample cue, as it held no information that could guide the subsequent response. During blocks of Match trials participants had to respond at the time of the probe by pressing the left button if the probe stimulus matched the sample stimulus or the right button if they did not match. Each block lasted 4.14 min.

##### Factor 2: stimulus type (line, face)

During both Respond and Match blocks the stimulus type was pseudo-randomly intermixed and could be either a gray line or a greyscale face. The faces were obtained from the Max Planck Institute for Biological Cybernetics database (Blanz and Vetter, 1999). For Line stimuli the participant was first presented with a horizontal line as a sample. At the time of the probe the participant was presented with a vertical line that was either the same or a different length. For Face stimuli, the participant was presented with a frontward facing face as a sample. At the time of the probe the face stimuli was presented at a 30° orientation facing either leftward or rightward (the presentation of leftward facing and rightward facing faces on the left or right of the screen was counterbalanced). This approach forced participants to encode stimulus identity and not just perceptual features of the stimulus.

The combination of these two factors with two levels each resulted in four conditions:

1. Line Respond: is the probe Line on the left or right of the screen? (40 trials)
2. Face Respond: is the probe Face on the left or right of the screen? (40 trials)
3. Line Match: is the length of the probe Line the same as the length of the sample Line? (40 trials)
4. Face Match: is the probe Face the same as the sample Face? (40 trials)

Participants practiced four to six shorter blocks of the task before entering the MRI scanner to make sure they understood the task. This typically lasted ∼7 min.

### Behavioral analyses

Behavioral measures were analyzed using a two way repeated measures ANOVA. Two factors of Task (Match, Respond) and Stimulus (Face, Line) were included with an additional between subject’s factor of group (young, old). This was used to assess differences in error rate, reaction time (RT), and RT variability. RT variability (intra-individual coefficient of variation) was calculated by dividing the RT standard deviation of each individual by their mean RT (Stuss et al., 2003; Bellgrove et al., 2004).

### Apparatus

Subjects lay supine in an MRI scanner with the thumb of the right hand positioned on a two-button MRI-compatible response box. Stimuli were projected onto a screen behind the subject and viewed in a mirror positioned above the subjects face. Presentation software (Neurobehavioral Systems, Inc., USA) was used for stimulus presentation both inside and outside the scanner. TTL pulses were also used to drive the visual stimuli in Presentation. Event timings and RTs were calculated off-line using event timings acquired by a separate laptop running Brain Recorder (Brain Products, Munich, Germany) at a higher sampling frequency (5000 Hz).

### fMRI data acquisition

We first acquired a high-resolution T1-weighted anatomical MPRAGE image (FOV = 230 mm, thickness = 0.9 mm, voxel size = 0.9 mm × 0.9 mm × 0.9 mm), followed by phase and magnitude images at different echo times (TE_1_ = 1.46 ms, TE_2_ = 7 ms), which were used to generate a voxel displacement map. Each participant then performed a single EPI session containing 1024 volumes lasting ∼34 min. The field of view covered the whole brain, 224 mm × 224 mm (64 × 64 voxels), 34 axial slices were acquired (0.05 mm slice gap) with a voxel size of 3.5 mm × 3.5 mm × 4 mm; TR = 2 s, TE = 32, flip angle = 78°. This was a sparse-sampling sequence with the slices compressed to the first 1700 ms of the TR, leaving 300 ms without gradient switching to facilitate the simultaneously recorded EEG (Debener et al., 2005). The combined EEG/fMRI data will be presented in a separate manuscript. All MRI data was collected on a Philips 3T Achieva MRI Scanner (Trinity College Dublin).

### fMRI pre-processing

Scans were pre-processed using SPM8^1^. Images were realigned and unwarped using field maps to correct for motion artifacts, susceptibility artifacts and motion-by-susceptibility interactions (Andersson et al., 2001; Hutton et al., 2002). Images were subsequently normalized to the ICBM EPI template using the unified segmentation approach (Ashburner and Friston, 2005). Lastly, a Gaussian kernel with a full-width at half-maximum (FWHM) of 8 mm was applied to spatially smooth the image.

### fMRI analyses

#### Group ICA analysis

A single group spatial ICA was run using the GIFT toolbox^2^. In this approach single-subject datasets were first compressed using principal component analysis (PCA, 123 components), single-subject data were then combined and PCA was performed for a second time on the whole group. Spatial ICA was then performed using the infomax algorithm (Bell and Sejnowski, 1995), with subsequent back reconstruction into single subjects (Calhoun et al., 2001; Erhardt et al., 2011). The resulting output is an independent component map and an associated timecourse for every component and subject. A modified minimum descriptive length (MDL) criteria (Li et al., 2007) determined that the optimal number of independent components was 82 and ICASSO was run with 100 re-runs and random initial conditions to ensure a robust decomposition (Himberg et al., 2004). Components with a quality (iQ; the difference between intra-cluster and extra-cluster similarity) below 0.9 were excluded from further analysis as were components that significantly correlated with regions of white matter or CSF. Head movement components (i.e., ringing around the edge of the brain) were also excluded from further analysis.

The Mancovan toolbox (Allen et al., 2011) was used to determine relationships between IC networks and descriptive variables such as age, gender, and task performance. This approach allowed us to investigate within component effects by analyzing IC spatial maps (SMs), and IC timecourse spectra as well as how descriptive variables modulate connectivity between networks using functional network connectivity (FNC; Jafri et al., 2008). For each component the BOLD spectrum were estimated on the detrended subject-specific timecourses (removing the mean, slope, and period π and 2π sines and cosines over each timecourse) using the multi-taper approach as implemented in Chronux^3^, with the time-bandwidth product set to three and the number of tapers set to five (Mitra and Bokil, 2008). These are the default settings within the Mancovan toolbox.

Two mancovan models were run which both included age, gender, neuropsychological measures (NART, Logical memory subtest of the WMS, MMSE), and head movement (rotation and translation). Task performance (accuracy and RT) was also included in these models, but RT values for line match and face match performance were highly correlated (*r* = 0.96, *p* = 2e−19). In order to improve model estimation we ran two separate models; (1) face match performance orthogonalized with respect to line match performance (FM_r), and (2) line match performance orthogonalized with respect to face match (LM_r). Two linear regressions were used to calculate these residual values. As such one model included the aforementioned variables along with face match accuracy (FM_acc), face match RT (FM_RT), residual line match accuracy (LM_r_acc) and residual line match reaction time (LM_r_RT), and a second model was run with residual face match accuracy (FM_r_acc), residual face match RT (FM_r_RT), line match accuracy (LM_acc) and line match reaction time (LM_RT).

Multivariate analyses were first performed in order to assess the extent to which each of the independent variables explained variance in the data (Figure 3). At this stage redundant variables that do not explain significant variance in the data (*p* > 0.05) are removed from the model. This procedure determines how well the independent variables explain variance within the dependent variables once other independent variables are taken into account. For example, Figure 3 shows that for component 58 BOLD spectral power is significantly modulated by FM accuracy, FM RT, gender, and rotation (*p* < 0.05, uncorrected). Importantly, we can see that rotation is the strongest predictor variable, explaining more variance in the BOLD spectrum then any other variables. Components will only be described as showing a significant relationship with task accuracy if they show the strongest relationship with BOLD spectral power based on these multivariate analyses.

In order to determine which spectral bins were associated with task performance we additionally performed univariate analyses. Partial correlation was used to measure the strength of the linear relationship between two variables [e.g., log(power) and face match accuracy] after adjusting for all other independent variables. Univariate tests were corrected for multiple comparisons at *p* < 0.05 using false discovery rate (FDR; Genovese et al., 2002).

#### Standard GLM analyses

Along with the ICA analyses we also conducted two standard GLM analyses implemented in SPM8 (Friston et al., 1995a,b). The first modeled events using the canonical hemodynamic response function (hrf), the second modeled events using Fourier basis functions (2 sine and 2 cosine functions of different frequencies with a 15-s Hanning window; Balsters and Ramnani, 2008). All first level models included nine event types. Sample and probe cues for each of the four conditions were modeled as eight separate event types. Trials in which responses were incorrect, too early (before the probe cue) or too late (responded after the presentation of the next sample cue) were modeled separately as a ninth event-type and differentiated from experimental conditions. This ninth event type included the onsets from both the sample and probe cues in error trials. Thus, activity time-locked to incorrect trials was excluded from regressors explaining instruction related activity. The residual effects of head motion were modeled as covariates of no interest in the analysis by including the six head motion parameters estimated during the realignment stage of the pre-processing. Prior to the study, a set of planned experimental timings were generated from two volunteers who performed the task outside of the scanner. These timings were carefully checked so that they resulted in an estimable GLM in which the statistical independence of the nine event types was preserved (piloting on volunteers allowed to generate a realistic error trial regressor).

To determine voxels significant at the group level, *t*-contrasts were incorporated into a random effects analysis using either one or two sample *t*-tests for the analyses using the canonical hrf or two way ANOVAs for analyses using the Fourier basis functions. ANOVAs had two factors; Group (two independent levels) and Basis functions (five non-independent levels). In all cases contrast images describing the main effect of stimulus (face vs. line), main effect of task (match vs. respond), and stimulus × task interactions at the single-subject level were calculated for both sample and probe cues. For analyses using the canonical HRF this was one contrast image per subject whereas analyses using the Fourier basis set used five contrast images per subject (one for each basis function).

Significant within group differences were established using a conjunction analysis (Price and Friston, 1997; Friston et al., 2005). This analysis confirms what is statistically similar across groups. Significant group differences were run on the same model. Beta values for the face match condition were also input into a one-sample *t*-test in order to see if beta values correlated with task accuracy in a similar manner to the ICA analyses. All results were corrected for multiple comparisons (FWE, *p* < 0.05).

## Results

### Behavior

#### Error rates

For both young and old groups there was a significant main effect of task [*F*(1, 28) = 170.99, *p* < 0.001] as significantly more errors were made in the matching task (7.46 error trials ± 0.53) compared to the respond task (0.58 error trials ± 0.12). Both groups also made significantly more errors for faces (4.88 error trials ± 0.28) compared to lines [3.41 error trials ± 0.35; *F*(1, 28) = 28.85, *p* < 0.001]. There was also a significant stimulus by task interaction [*F*(1, 28) = 34.42, *p* < 0.001] as significantly more errors in face match condition than any other condition.

**Figure 2:**
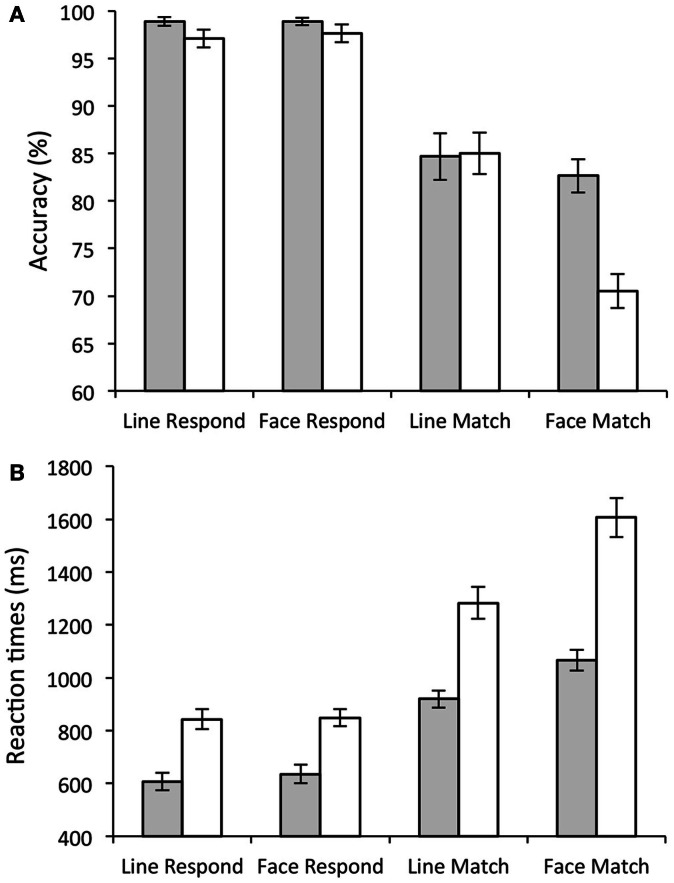
**Behavioral Results**. Bar graphs showing task accuracy **(A)** and response times. **(B)** Gray bars show average scores for young participants; white bars show average scores for elderly participants. Error bars show the standard error.

A number of group differences were also present. Although the main effect of group [*F*(1, 28) = 3.77, *p* = 0.06] did not reach significance, there were clear selective deficits in the performance of old participants compared to young. This was seen in the significant group × stimulus interaction [significantly more errors to faces than lines in the old participants; *F*(1, 28) = 16.84, *p* < 0.001], and a significant group × task × stimulus interaction [*F*(1, 28) = 21.05, *p* < 0.001], as elderly participants made significantly more errors in the face matching condition compared to any other condition [*T*(1, 28) = 4.19, *p* < 0.001]. This suggests that key difference in performance between the young and old participants was in the face match condition (see Figure 2A).

#### Reaction time

As with error rate, all participants showed a significant main effect of task on RT (slower RTs during match (1236.99 ± 43.33 ms) compared to respond conditions [718.2 ± 24.54 ms; *F*(1, 28) = 159.85, *p* < 0.001]. There was also a significant main effect of stimulus type [slower to respond to faces (1044.08 ± 30.92 ms) compared to lines (911.11 ± 26.99 ms); *F*(1, 28) = 189.73, *p* < 0.001], and a significant stimulus × task interaction [significantly slower on face matching compared to all other conditions; *F*(1, 28) = 142.92, *p* < 0.001].

Older participants showed significantly slower RTs compared to young participants (Old (1148.25 ± 41.8 ms); Young (806.93 ± 39.1 ms); significant main effect of group [*F*(1, 28) = 35.564, *p* < 0.001]). There were also significant group × stimulus interactions [*F*(1, 28) = 22.14, *p* < 0.001; old participants were significantly slower than young participants to respond to faces compared to lines] and significant group × task interactions [*F*(1, 28) = 12.86, *p* < 0.005; Older participants were significantly slower than young participants to match compared to respond]. Finally there was also a significant group × task × stimulus interaction illustrating the significant difference in face matching in young compared to old [*F*(1, 28) = 34.56.2, *p* < 0.001]. These results are illustrated in Figure 2B.

Whilst there were no significant group effects on RT variability there was a significant main effect of stimulus type [*F*(1, 28) = 8.49, *p* < 0.01] on RT variability (greater variability for line stimuli compared to faces) and a significant task × stimulus interaction [*F*(1, 28) = 4.3, *p* < 0.05; greater variability in the line match condition compared to all other conditions].

### fMRI analyses

#### Group ICA analyses

**Figure 3:**
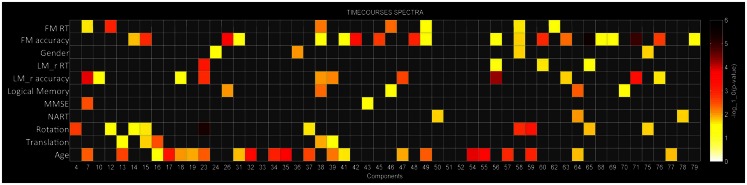
**Multivariate statistics**. Results from the reduced mancova models, depicting the significance of covariates of interest and nuisance predictors for power spectra in log_10_(*p*) units. Gray cells indicate terms that were removed from the full model during backward selection process.

Out of 82 ICs, 54 were included in the mancovan models. Figure 3 shows the strength of the relationship between spectral power for each IC and each of the variables of interest and nuisance variables. ICs were ignored if they showed a stronger relationship with head movement than task performance.

##### Task performance

**Figure 4:**
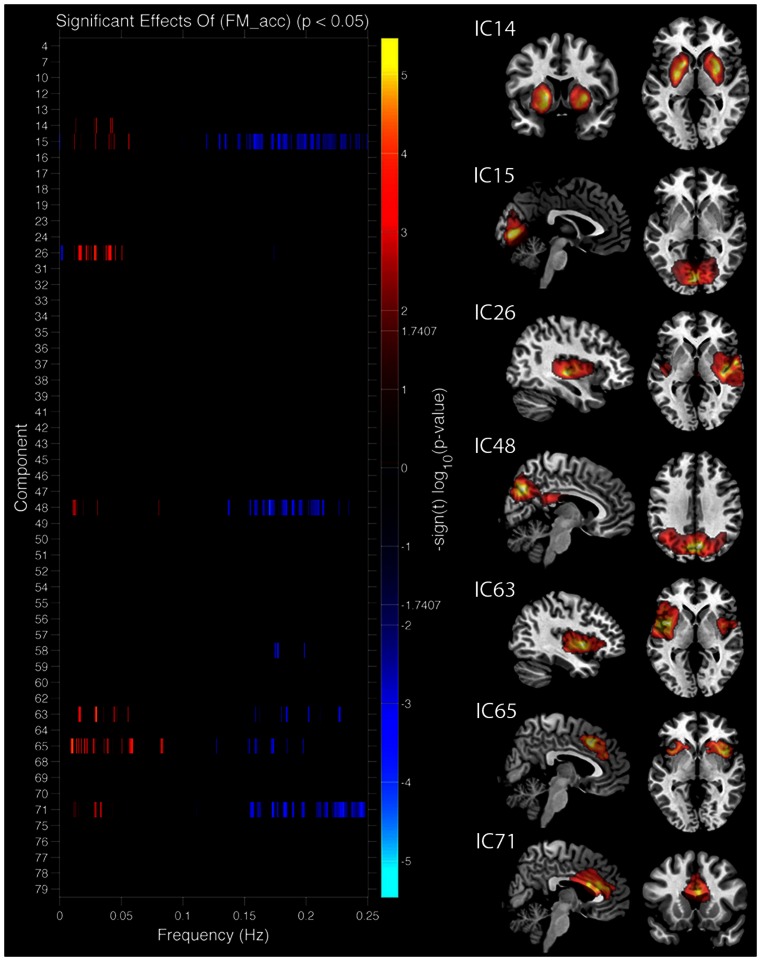
**Components showing a relationship between spectral power and face match accuracy**. Left column shows components where spectral power significantly covaried with task accuracy. Red markers indicate a positive relationship with task accuracy (greater spectral power with higher accuracy), blue markers indicate a negative relationship (greater spectral power with lower accuracy), black indicates their was no significant difference after correcting for multiple comparisons. Right column shows spatial maps for components which showed a significant relationship with face match accuracy. All results are FDR thresholded (*p* < 0.05).

We first examined the relationship between IC features and accuracy. SMs and FNC showed no significant relationship to task accuracy, but for a number of ICs BOLD spectral power was significantly correlated with task performance (see Figure 4). In all cases there was a positive relationship between 0 and 0.1 Hz BOLD spectral power and task performance (greater spectral power = better performance) and a negative relationship between 0.1 and 0.25 Hz power and accuracy (greater spectral power = poorer performance). Spectral power within the anterior cingulate cortex (ACC) (area 24; IC 71) correlated to both LM and FM accuracy. Spectral power within the caudate nuclei (IC 47) was specific to LM accuracy. Spectral power within six networks related to FM accuracy including putamen (IC 14), visual (IC 15), right superior STG (IC 26), precuneus (posterior DMN; IC 48), insular (IC 63), and the salience network (SN) (ACC and bilateral anterior insular; IC 65) (see Table 1 for details). All of these results were significant in the analysis of the residual values (FM_r_acc and LM_r_acc) as well as analysis of the original values. Table 2 shows the results of linear multiple regression using single-subject IC timecourses as the dependent variable and the GLMs used for the hrf analysis as independent variables (see Standard GLM Analyses above for details). A one-sample *t*-test was performed on beta values to establish if there was a significant relationship between event timecourses and IC timecourses (*p* < 0.05, uncorrected). Two sample *t*-tests were also run on these same beta values to establish whether the relationship differed between groups (*p* < 0.05, uncorrected).

**Table 1:** **Peak activations of spatial maps showing a relationship with task performance**.

Component # (Iq)	Brain region	Cluster size	*t*	Co-ordinates			Cytoarchitectonic BA (probability if available)
				*x*	*y*	*z*	
**FACE AND LINE MATCH ACCURACY**							
71 (0.93)	Left anterior cingulate cortex	3091	26.91	0	32	14	Area 24
**FACE MATCH ACCURACY**							
14 (0.98)	Left putamen	3277	31.42	−18	8	0	n/a
	Right putamen	3123	29.83	28	4	0	n/a
15 (0.98)	Left calcarine gyrus	6131	46.69	−2	−84	−2	Area 17 (90%)
26 (0.98)	Right insula lobe	4400	27.11	38	−20	0	Insula (Ig2) (90%)
	Left superior temporal gyrus	188	10.77	−46	−12	−2	Insula (Ig2) (10%)
48 (0.97)	Right precuneus	6738	32.23	6	−70	34	SPL (7M) (60%)
	Left angular gyrus	Same cluster	16.14	−38	−60	40	hIP2 (10%), hIP3 (10%)
	Right angular gyrus	Same cluster	10.86	36	−58	42	hIP1 (20%), hIP3 (10%)
63 (0.97)	Left insula lobe	3811	25.19	−40	4	0	Area 48
	Right insula lobe	1083	16.6	42	0	6	Area 48
	Right angular gyrus	319	11.41	52	−56	26	IPC (PGa) (50%)
	Left supramarginal gyrus	224	9.06	−58	−34	28	IPC (PF) (90%)
	Right anterior cingulate cortex	93	7.86	4	16	28	Area 24
65 (0.97)	Right superior medial gyrus	1495	24.05	4	20	42	Area 32
	Right insula lobe	1209	21.1	40	10	−2	Area 48
	Left insula lobe	563	17.9	−36	16	−10	Area 48
**LINE MATCH ACCURACY**							
47 (0.98)	Right caudate nucleus	3655	29.34	8	18	2	n/a
	Left caudate nucleus	Same cluster	27	−8	16	0	n/a
**FACE MATCH RT**							
46 (0.98)	Left inferior temporal gyrus	1121	18.89	−48	−62	−6	Area 37
	Right inferior temporal gyrus	862	16.13	46	−60	−14	Area 37
	Left cerebellum	445	17.29	−4	−78	−12	HVI (6%)
	Right superior parietal lobule	180	12.18	24	−72	48	SPL (7P) (40%)
	Left precuneus	152	11.11	−4	−52	18	Area 30
	Left middle cingulate cortex	90	9.8	−2	14	38	Area 24
62 (0.97)	Left anterior cingulate cortex	4681	30.48	−6	42	20	Area 32
	Left inferior frontal gyrus (p. orbitalis)	295	13.95	−48	24	−14	Area 47

*The quality index (Iq) associated with each RSN is listed in parentheses adjacent to the component number. Cluster size refers to the number of voxels in each cluster, negative *x* co-ordinates refer to left hemisphere activations. Cytoarchitectonic probabilities were established where possible by using the Anatomy toolbox (Eickhoff et al., 2005, 2006, 2007)*.

**Table 2:** **Linear regression between event-related task timecourses and IC timecourses**.

IC	Sample line respond	Sample face respond	Sample line match	Sample face match	Probe line respond	Probe face respond	Probe line match	Probe face match
**1 SAMPLE *t*-TEST**								
14 (BG)		Y			Y	Y	Y	Y
15 (Visual)	Y	Y	Y		Y	Y	Y	Y
26 (Left STG)								Y
48 (Posterior DMN)					Y	Y	Y	Y
63 (Insula)					Y	Y	Y	Y
65 (Salience)	Y	Y	Y				Y	Y
71 (ACC)					Y	Y	Y	Y
46 (Fusiform)	Y	Y	Y	Y		Y	Y	Y
62 (ACC)	Y		Y	Y	Y	Y		Y
**2 SAMPLE *t*-TEST**								
14 (BG)					Y	Y		
15 (Visual)					Y	Y	Y	Y
26 (Left STG)	Y	Y				Y		
48 (Posterior DMN)			Y			Y		
63 (Insula)					Y			
65 (Salience)		Y					Y	
71 (ACC)							Y	
46 (Fusiform)	Y			Y		Y		
62 (ACC)	Y	Y					Y	Y

*Y indicates a significant relationship (as measured by beta values) between task regressors and IC timecourses (1 Sample *t*-test) or a significant difference between groups (2 Sample *t*-test). Significance thresholded at *p* < 0.05 uncorrected*.

**Figure 5:**
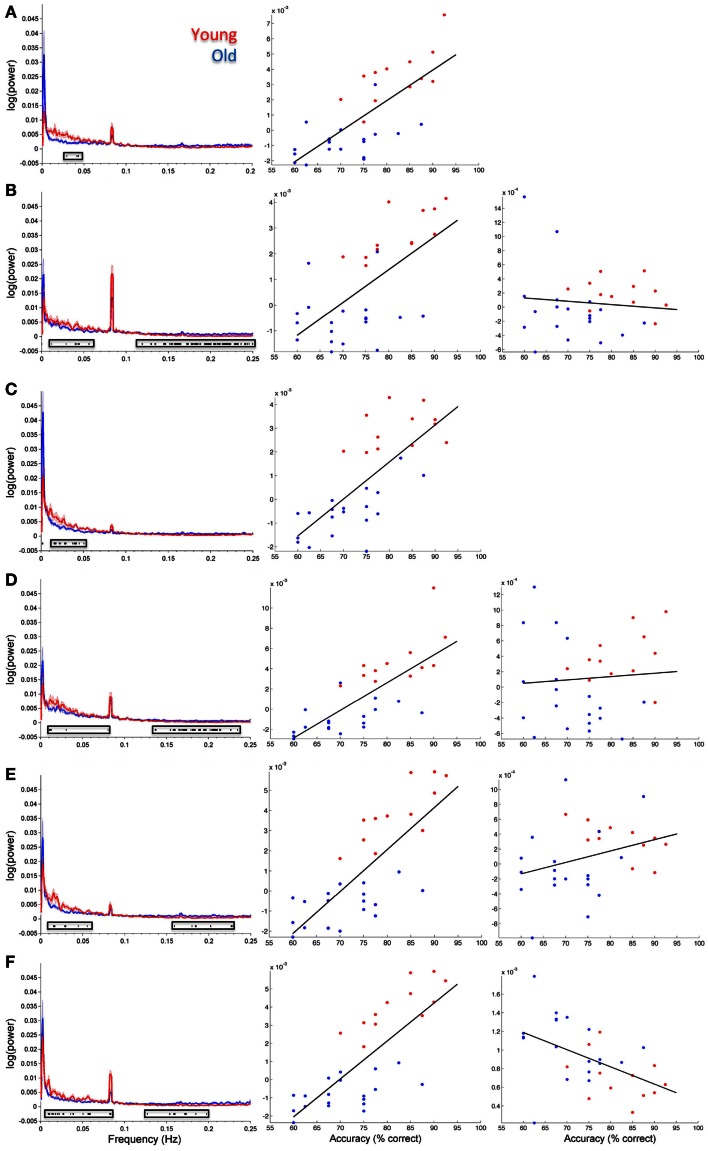
**Group spectral profiles and correlations with face match accuracy**. Left column shows spectral power distributions for young and old participants. Shaded error bars show the standard error. Black markers underneath highlight where spectral power covaried with task accuracy (these are the same values shown in Figure 4). Middle and right columns show correlations with spectral power and accuracy after age was regressed out of the data. Middle column shows correlations for significant frequency points at lower frequencies (<0.1 Hz). The right column shows correlations for significant frequency points >0.1 Hz. **(A)** Putamen (IC 14), **(B)** Visual cortex (IC 15), **(C)** right STG (IC 26), **(D)** precuneus (posterior DMN; IC 48), **(E)** left insula (IC 63), **(F)** cingulo-insula network (salience network; IC 65). In all plots red refers to young participants and blue to elderly.

Figure 5 shows spectral profiles for both young and older participants and the correlations between spectral power and accuracy after variance associated with age had been removed from the data. Even after age-related variance was removed from the data there were still very strong correlations between task accuracy and spectral power below 0.1 Hz (*r* values between 0.64 and 0.79). However, removing age-related variance from higher frequencies (>0.1 Hz) typically removed the relationship between spectral power and accuracy for most ICs. Only the SN (IC 65) maintained significance at higher frequencies after removing age-related variance. All of the BOLD spectra presented in Figure 5 show a clear peak at 0.08 Hz (every 12.5 s). This peak reflects the presentation of the stimuli and is not an artifact. Resting state data acquired immediately prior to the collection of this task was run through a similar analysis pipeline and the 0.08-Hz peak was not present (Balsters et al., 2013). Table 3 shows partial correlation values for BOLD spectral power and task accuracy after age-related variance was regressed out of the data. Partial correlations were run across all subjects as well as young and old subjects only.

**Table 3:** **Partial Correlations between BOLD frequency power and task performance with age-related variance removed**.

IC	Low frequency (0–0.1 Hz)						High frequency (0.1–0.25 Hz)					
	Group		Young		Old		Group		Young		Old	
	*r*	*p*	*r*	*p*	*r*	*p*	*r*	*p*	*r*	*p*	*r*	*p*
14 (BG)	0.7451	**<0.001**	0.6869	**0.0136**	0.4522	0.0519						
15 (Visual)	0.6414	**0.001**	0.6414	**0.001**	0.0655	0.79	−0.2419	0.1898	−0.2833	0.3722	−0.3849	0.1037
26 (Left STG)	0.8085	**<0.001**	0.0962	0.7662	−0.1585	0.5168						
48 (Posterior DMN)	0.7468	**<0.001**	0.627	**0.0291**	0.5326	**0.0189**	−0.293	0.1097	−0.219	0.4941	−0.5345	**0.0184**
63 (Insula)	0.7695	**<0.001**	0.7959	**0.002**	0.5348	**0.0183**	−0.3017	0.09	−0.6288	**0.0285**	−0.4624	**0.0462**
65 (Salience)	0.758	**<0.001**	0.7899	**0.022**	0.5322	**0.019**	**−0.4991**	**0.0042**	−0.6403	**0.0249**	−0.4347	0.0629

**p-*Values marked in bold were significant (*p* < 0.05, uncorrected)*.

**Figure 6:**
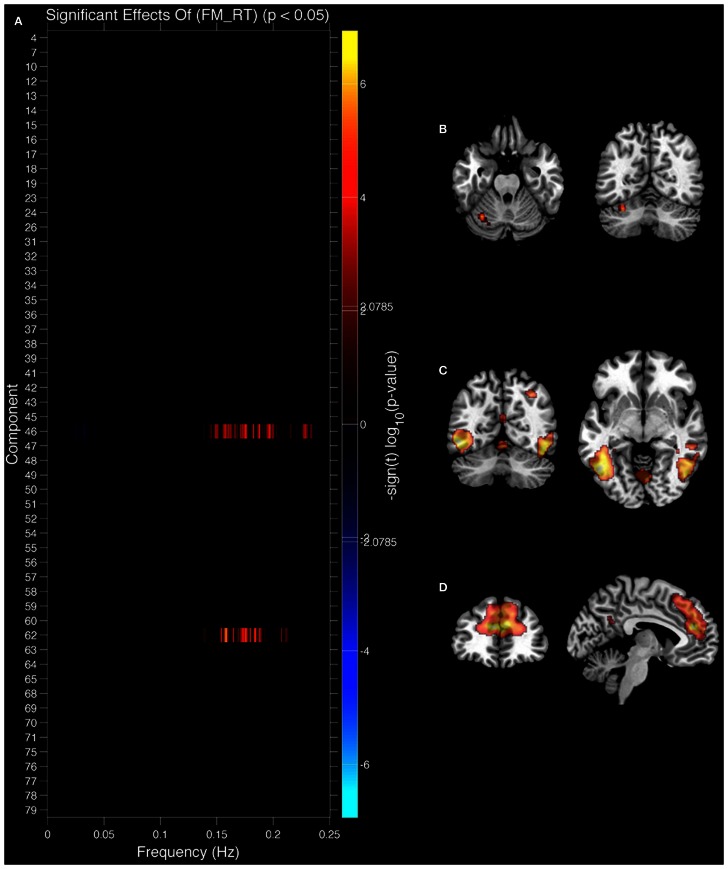
**Components showing a relationship between spectral power and face match reaction time (RT)**. **(A)** Components where spectral power significantly covaried with face match RT. Red markers indicate a positive relationship (greater spectral power = slower RT), black indicates their was no significant difference after correcting for multiple comparisons. **(B)** Significant covariation with voxel intensity and face match RT within left cerebellar lobule HVI. **(C)** IC 46 spatial map. **(D)** IC 62 spatial map. All results are FDR thresholded (*p* < 0.05).

We also analyzed the extent to which RT related to IC features (see Figure 6). In this case only the original values explained IC features and there were no significant effects of residual values (FM_r_RT or LM_r_RT). Both FM_RT and LM_RT were significantly correlated to SM activity within motor lobules of the cerebellum [left lobule HVI (85%) (Diedrichsen et al., 2009)]. LM RT was correlated with 0.15–0.2 Hz spectral power in the thalamus [IC 12, Visual Thalamus (Behrens et al., 2003)], and FM RT was correlated with 0.15–0.2 Hz spectral power in fusiform gyrus (IC 46) and ACC (area 32; IC 62) (see Table 1 for details). In all three cases 0.15–0.2 Hz spectral power was positively correlated with RT (greater spectral power = slower RT). The relationship between spectral power and RT was not present after variance associated with age had been removed from the data.

#### GLM analyses

##### Faces vs. lines

Within group analyses showed significant activations in predicted regions. For example, a comparison of stimulus type (faces vs. lines) showed greater activity in bilateral fusiform gyrus for faces compared to lines. This was present both at the time of the sample and probe cue. However, there were no significant group differences. The FDR thresholded main effect of faces vs. lines was compared spatially with all the ICs found to correlate with task performance by overlaying these images in MRIcron. There was no spatial overlap between any of these ICs and the main effect of stimulus type. These results were consistent for HRF and Fourier models.

##### Match vs. respond

**Figure 7:**
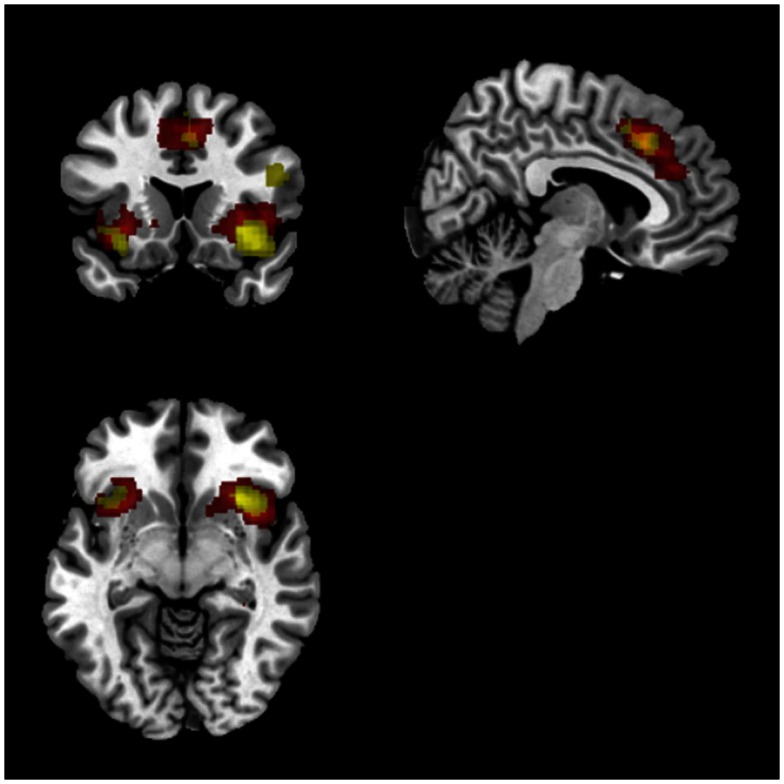
**Overlap between IC 65 (salience newtork) and SPM results**. Red voxels show the spatial map for IC 65 which was identified to track task accuracy at both low (<0.1 Hz) and high (>0.1 Hz) frequencies. Activations in yellow were from the SPM analysis showing common activations between both groups for match compared to respond blocks. Both sets of activations were FDR corrected (*p* < 0.05).

Similarly, a comparison of task (match vs. respond) showed greater activation in right middle/inferior frontal gyrus, as well as ACC and bilateral insula for match compared to respond. Overlaying this FDR thresholded activation map with ICs found to correlate with task performance showed a clear spatial overlap with the previously identified SN (IC65; see Figure 7). There was no spatial overlap with any other of the ICs found to correlate with task performance. Despite significant behavioural differences (group × task interaction) there were no significant group differences for this comparison. These results were consistent for HRF and Fourier models.

##### Stimulus × task interaction

There were no significant within- or between group activations for the stimulus × task interaction, despite there being very significant behavioral differences. These results were consistent for HRF and Fourier models.

##### Face match and task performance

In order to more directly compare the ICA and GLM analyses, we performed a one-sample *t*-test looking for correlations between accuracy and beta values associated with Face match condition (both sample and probe). There were no significant correlations.

## Discussion

It has been repeatedly shown that elements of executive function, such as working memory, degrade with age (Grady and Craik, 2000). As in other studies (Grady et al., 1995, 1998) we found that elderly participants performed significantly worse than young controls on a DMS task (both in terms of error rate and RT), with the group difference being largest when matching facial stimuli (see Figure 2). Whilst standard GLM-based approaches failed to distinguish between age groups or task performance, a combination of ICA and multi-taper spectral analyses illustrated a number of functional networks where BOLD spectral power tracked task performance. Multivariate statistics further demonstrated that task accuracy was the strongest predictor variable for BOLD spectral power within these networks, stronger than age, head movement, gender, or any neuropsychological variables (Figure 3).

### Age-related changes in functional networks during DMS performance

The functional networks identified as tracking task performance regardless of age included the primary visual network, temporal-parietal network, posterior default-mode network, SN, and basal ganglia. The visual, posterior DMN, and SNs also showed higher frequency BOLD oscillations that negatively correlated with both task accuracy and age. The differences between high and low frequency BOLD oscillations will be discussed below. Studies using the delayed match-to-sample task have typically found increased activity within the frontal-parietal network (FPN) and decreased activity within the DMN (Grady et al., 2010; Spreng et al., 2010; Salami et al., 2012). When investigating aging populations it has been further shown that the DMN decreases less during task performance with age whilst the FPN increases with age (Grady et al., 2010; Salami et al., 2012). There has been some indication that this increased FPN activity is compensatory, whilst others argue that this may indicate reduced neural efficiency (see Grady, 2012 for review). The results of this study move the focus away from prefrontal regions in working memory and place a greater emphasis on the DMN. It is well established that DMN connectivity decreases with age during rest (Damoiseaux et al., 2008; Allen et al., 2011; Balsters et al., 2013), however there is more debate surrounding DMN connectivity during task performance. Whilst some studies have shown increased DMN activity during task compared to young controls (Grady et al., 2010) others have shown a continued decrease in DMN functional connectivity (Andrews-Hanna et al., 2007; Sambataro et al., 2010). Sambataro et al. (2010) scanned young and old participants during a working memory task (1- and 2-back tasks) and showed reduced DMN connectivity with age, and that increased connectivity within this network was correlated with better performance. In line with the results of this study the Sambataro et al. (2010) also showed reduced low frequency BOLD spectral power (0.03–0.08 Hz) in the posterior DMN related to age and increased BOLD spectral power within the same band limits as task difficulty increased. Garrett et al. (2013) also found reduced BOLD variability with aging in regions of the posterior DMN during task performance (including DMS task) compared to rest. The precise role of the DMN in cognitive control is unclear, however these findings add to previous suggestions that the posterior nodes of the DMN are involved in memory retrieval (Menon, 2011; Vannini et al., 2011).

The SN was the only network which showed both low and high BOLD frequency correlates of task accuracy after accounting for age-related variance (Figure 5F). The SN comprises of bilateral anterior insula and ACC. The insula has been shown to be an important node in functional connectivity, linking multiple brain regions, and functional networks (see Menon and Uddin, 2010 for review). Two of the key roles proposed for the SN are: (1) detection of salient events and (2) switching between large-scale functional networks once a salient event has been detected (Menon and Uddin, 2010; Menon, 2011). Along with being the only IC to track accuracy at both high and low BOLD frequencies, this was also the only IC to overlap with GLM-based results (match > respond). As in our study, Sridharan et al. (2008) found a strong overlap between the SN found using ICA and GLM-based analyses. The behavioral results of our study showed a strong effect of task on RTs and accuracy (poorer performance on match trials compared to respond trials) indicating that the match task was more difficult. It is therefore likely that increased attentional demands were placed on the match blocks compared to the respond blocks, thus highlighting the SN in the GLM analyses for match > respond events. Control signals from the SN are believed to have a top-down influence on multiple networks including basic sensory networks and functionally complex networks like the DMN and FPN. It is possible that control signals from the SN were impacting on BOLD oscillations within other identified networks such as the posterior DMN and visual cortex, however we did not find a significant correlation between these networks after correcting for multiple comparisons. One would also predict based on previous studies that the SN signal would elevate activity within the FPN rather than the DMN. This may suggest that an increase in FPN connectivity is not directly correlated with task accuracy in aging and may indeed index inefficient neural activity. As mentioned previously, there is still a great deal of debate about whether increased FPN connectivity is a positive or negative marker of executive function in aging (Grady, 2012).

### Multiple BOLD frequencies differentially contribute to task performance

Our results suggest two broad relationships exist between task accuracy and BOLD oscillations; power at BOLD frequencies below 0.1 Hz were positively correlated with working memory performance and unrelated to the age of the subjects, whilst power at frequencies above 0.1 Hz were negatively associated with task performance and typically contained age-related variance (the SN being the only exception). Previous studies have also shown that multiple oscillatory dynamics are contributing to low frequency fluctuations in the BOLD signal and that these different oscillations may have distinct functional roles (Salvador et al., 2008; Baria et al., 2011; Niazy et al., 2011). Studies by Garrity et al. (2007), Malinen et al. (2010), and Calhoun et al. (2011) have shown that control groups had stronger BOLD fluctuations below 0.05 Hz whilst patient groups (schizophrenic, bipolar, and chronic pain patients) had stronger high frequency BOLD fluctuations (>0.1 Hz). Similarly, Allen et al. (2011) showed decreasing BOLD frequency power (<0.15 Hz) with age, whilst some RSNs showed increasing spectral power with age at frequencies greater than 0.2 Hz. All of these studies would suggest that increased higher frequency BOLD oscillations, present in schizophrenic patients, bipolar patients, chronic pain patients, and healthy aging, are a negative symptom (although none of these studies directly linked higher frequency oscillations to behavioral or neuropsychological measures). Our results are in keeping with the idea that high frequency BOLD fluctuations are a negative symptom given that we find a negative correlation with working memory performance and high frequency BOLD spectral power. One difference between this study and the studies of Garrity et al. (2007), Malinen et al. (2010), and Calhoun et al. (2011), is that our data was collected during task performance whilst the other studies report used resting data. Although it is likely that differences in the underlying causes of BOLD oscillations will differ between rest and task, Calhoun et al. (2008) showed that decreased low and increased high frequency BOLD spectral power was present in the same schizophrenic patients during both task performance (auditory oddball) and rest.

It has been proposed by Garrity et al. (2007) and Malinen et al. (2010) that increased higher frequency oscillations might be indicative of reduced connectivity within the functional network. It is well established that both structural and functional connectivity decreases with age (Andrews-Hanna et al., 2007; Damoiseaux et al., 2009; Allen et al., 2011), therefore an increase in BOLD spectral power at higher frequencies may represent reduced network synchronization. Cohen (2011) had participants perform a similar working memory task and investigated the delay period between the sample and probe using EEG. Cohen (2011) found a significant negative relationship between performance and peak oscillatory frequency (faster oscillations = poorer performance) during the delay period. Peak oscillatory frequency was also strongly negatively correlated with the structural connections between the hippocampus and ventrolateral PFC. These results add to the evidence that slower frequencies are necessary for encoding and maintaining complex information (Cohen, 2011; Honey et al., 2012), whilst changes in higher frequency oscillations might be indicative of reduced functional and structural connectivity.

A number of previous studies have suggested that resting state BOLD fluctuations >0.1 Hz are noise (Wise et al., 2004; Birn et al., 2006; Zou et al., 2008; Zuo et al., 2010), and might reflect cardiac or respiratory signals. One must therefore ask whether the >0.1 Hz effects seen in this study might be related to cardiac or respiratory signals. Unfortunately, we did not collect cardiac or respiratory recordings so we can not completely rule out this possibility, but we would argue based on previous resting state studies that BOLD fluctuations >0.1 Hz can contain meaningful information. First, it has been shown that ICA is capable of isolating physiological noise sources from functional networks (Birn et al., 2008; Beall and Lowe, 2010; Allen et al., 2011). By excluding 28 components that correlated with white matter and CSF, displayed ringing around the edge of the brain, or had a variable decomposition, we believe we have managed to remove some physiological noise sources. Similar studies to ours were able to assess the impact of cardiac and respiration signals on BOLD oscillations at rest, and in both studies their results were not explained by these noise sources (Malinen et al., 2010; Baria et al., 2011). However, we would also reiterate that the strongest relationship between task accuracy and BOLD spectral power was at frequencies below 0.1 Hz that are widely acknowledged to reflect underlying neural fluctuations (He et al., 2010; He, 2011; Honey et al., 2012).

### Advantages and disadvantages of ICA/spectra approach compared to GLM approaches

A number of studies have previously demonstrated that BOLD signal correlates with task performance (Pessoa et al., 2002; Todd and Marois, 2004; Nagel et al., 2011). However, we believe there are a number of advantages to using BOLD frequency power instead of GLM-based values such as beta values or percent signal change. As mentioned previously, fluctuations in the BOLD signal are composed of a number of different oscillatory signals (Zuo et al., 2010; Baria et al., 2011; Niazy et al., 2011). As such, just investigating one oscillatory signal may not capture the underlying complexities that exist within BOLD data. Although BOLD variability has been shown to be more sensitive than mean BOLD signal, this approach still fails to take into account different BOLD frequency bands. For example, Garrett et al. (2013) found there was very little difference in BOLD variability within the elderly population between fixation and delayed match-to-sample performance. By investigating the entire BOLD spectrum we were able to find BOLD fluctuations that significantly correlate with delayed match-to-sample performance across young and old participants, as well as additional BOLD dynamics that are related to age. We therefore believe that this approach is more sensitive to brain-behavior relationships than other approaches such as GLM-based approaches and BOLD mean/variability measurements.

It may also be possible to integrate the spectral analyses conducted within this study with GLM approaches. For example, one could apply this spectral analysis to regions identified using a GLM approach instead of using ICA timecourses. However, GLM-based approaches require additional assumptions about the hrf. A number of studies have shown that BOLD response is far from canonical, changing across brain areas (Handwerker et al., 2004; Eichele et al., 2008; Wall et al., 2009), subjects (Aguirre et al., 1998), clinical populations (Rombouts et al., 2005), and in healthy aging (D’Esposito et al., 1999). In this study we used both the canonical HRF as well as more flexible Fourier basis functions to model events. The results were consistent across both GLM approaches, and neither of these highlighted the results established using ICA/spectral approaches. However, even if one uses multiple basis functions, or generates a custom HRF per subject, this still assumes that response functions are consistent from trial-to-trial. In event-related designs such as the one used in this study there is likely to be a great deal of trial-to-trial variability. By analyzing the spectral content of the whole time course we overcome this issue. However, this is also the main disadvantage of this approach. By analyzing the entire timecourse of the experiment we are not able to establish whether these BOLD spectral changes are time locked to specific cue types or task phases. Early investigations into working memory changes with age using the delayed match-to-sample task found that the deficit was specifically at the time of encoding rather than at the recognition/decision phase (Grady et al., 1995). Unfortunately, we are not able to address this question regarding the encoding and recognition phases of the experiment. It is possible to perform temporal regression on IC timecourses as we have done in this study (Table 2). However, this requires us to make assumptions about the shape of the hrf and trial-to-trial variability, which for reasons mentioned above may not be valid. Another alternative would be use a block design experiment where spectral content of encoding and recognition phases can be analyzed separately. Recent studies by Allen et al. (2012), Smith et al. (2012), and Sakoglu et al. (2010) are also investigating changes within and between functional networks over time. A modified version of these approaches may also allow us to investigate BOLD spectral changes in an event-related manner.

It is possible that the experimental design used in this study favored ICA/spectral analyses and biased against GLM approaches, however, we do not believe this to be the case. In this study we collected a long timeseries of data (∼34 min) which consisted of long 4.14 min blocks of task performance. Such a design is certainly amenable for Fourier transforms, however we do not believe that this unfairly biases against GLM approaches. Long, single session acquisitions such as the ones used in this study are recommended by a number of fMRI papers (Josephs and Henson, 1999; Smith et al., 2005, 2007). In addition the results of our study are consistent with previous studies of BOLD oscillations/variability conducted by Salvador et al. (2008) and Garrett et al. (2011) who used much shorter task blocks of 48 and 36 s (DMS task) respectively for their analyses. It may still be the case that the experimental design used in this study is inefficient for GLM approaches, both to identify significance and accurately model response magnitude. Different filtering procedures were also used in the ICA analysis compared to the GLM-based analyses which could have impacted on the results. However, given the consistency with previous studies (Garrett et al., 2010, 2011, 2013; Samanez-Larkin et al., 2010; Baria et al., 2011), we believe that ICA/spectral analyses are tapping into brain-behavior relationships that are lost in GLM approaches.

## Conclusion and Future Directions

This study demonstrates for the first time that BOLD spectral power is a useful index of brain-behavior relationships that appears to be more sensitive than traditional GLM approaches. Unfortunately the sluggish nature of the BOLD response does not make it possible to directly compare BOLD spectral measures with similar EEG and MEG spectral measures. Simultaneous EEG/fMRI studies have begun to investigate the relationships between EEG frequency bands and fMRI SMs (Mantini et al., 2007; Balsters et al., 2011, 2013), however in order to understand the relationship between M/EEG oscillations and BOLD oscillations one must contend with the fact that M/EEG oscillations are significantly faster than events used in a task paradigm whereas BOLD oscillations are more likely to be directly influenced by the task paradigm. Further research is necessary to establish (a) the potential relationships between EEG and fMRI frequency bands and (b) the reliability of >0.1 Hz BOLD fluctuations in both task and rest.

## Conflict of Interest Statement

The authors declare that the research was conducted in the absence of any commercial or financial relationships that could be construed as a potential conflict of interest.

## Footnotes

^1^www.fil.ion.ucl.ac.uk/spm

^2^http://mialab.mrn.org/software/gift

^3^http://chronux.org
